# Pensions and Health

**Published:** 1924-01

**Authors:** 


### HOSPITAL and HEALTH REVIEW

### PENSIONS AND HEALTH.

THAT medicine knows no means of prolonging life equal to the possession of a pension or annuity is one of the most notorious facts of life. Give a man a pension, even though it be small, and the inevitable churchyard recedes from the foreground, or it may be the middle distance, into the mists of futurity. It is clear, therefore, that pensions and vigorous health are closely linked. Even the military or naval pensioner, notwithstanding that he may have suffered from wounds, exercises a blunting effect on the scythe of Time—the Chelsea pensioner is a famous case in point. The reasons are so obvious and familiar that they hardly need stating—the peace of mind which comes from an assured, if narrow, future, and the absence of the hardships which too often fall upon unprotected old age. The Great War, as we have just been reminded by the issue of the sixth annual report of the Ministry of Pensions, has produced a new type of naval and military pensioner in the shape of the essentially civilian man who, by conscience or by force, became a soldier, or of his widow and children. It is startling and tragic, indeed, to learn that two men out of every five who enlisted have received compensation in some form from the Ministry of Pensions. To the end of last March we had already spent four hundred and sixty millions in this way, not one penny of which has ever been grudged by a grateful country. There were then two and a half millions of beneficiaries, including the wives, widows and children of men killed or disabled. The charge is, however, rapidly diminishing, and nine months ago the number of beneficiaries was a million fewer than when the total was at its peak. Men, women and children die, widows re-marry, and children pass beyond the pensionable age.

We have, however, done more than pension men who have suffered in limb or in function from the wounds and hardships of war. We have endeavoured so to mend their bodies and give ease to their minds that, in addition to their allowances from the State, they may be enabled to earn their living with a considerable measure of fullness, and we trust that there will never be any serious tendency so to restrict their pensions that needless pecuniary anxiety will enter into their lives. They deserve all that their country can do for them, and it is our obvious duty to smooth their path to the utmost, and to make it as easy as may be that they may enjoy relative comfort in old age, as well as health in the meantime. What is good for the old age of soldiers, even when they are only temporary soldiers, is good for all other classes of the community. Old age pensions, rather harshly conditioned as they are in regard to other income, have not only comforted the disabilities of age, but have unquestionably helped to reduce the death-rate and to lengthen the expectation of life. Until the Saturnian period comes there must always be many men and women who, for one reason or another, are unable to make provision for old age, but it should be one of the aims of true statesmanship to encourage every practicable means of making pensions more general. In a well-organised State (there has never been one yet) every occupation would have its pension system created and preserved by the co-operation of employer and employed. There are such systems already, no doubt, but they are relatively few in number. Some great firms have earned honourable distinction by placing themselves in the van of the movement for providing pensions for the staffs of all grades. These retiring allowances are a powerful incentive at once to thrift and to that self-respect which is one of the most precious possessions of mankind.

We observe with pleasure that Messrs. Cadbury, one of the great firms to which we have referred, are engaged upon the completion of their pension schemes by forming a fund to provide pensions for the widows of their employees. The fund will be fed by equal contributions from the company and the workers, and it will be of such dimensions that it will give a widow an allowance of one half the pension paid to her husband. This is not only philanthropy but good sound business. A man who knows that should he die before his wife, she will be entitled to half his pension, is likely to be a stronger, healthier, and more cheerful man, a better worker and the possessor of a more contented mind than one who has no such assurance and is constantly worried about the future of those dependent upon him. The rush and swirl of modern life are so great that the influence upon health of an easy mind is likely every year to grow more important. This is one reason the more why the need for the creation of pension systems for occupations should be kept firmly before the public mind. There is no more pitiful spectacle than an old age of poverty and want, especially when the sufferer cannot be accused of lack of duty to himself, his family, and the State. It is self-evident that an easy old age is not only likely to be a prolonged old age, but that both men and women who have not been tormented by fear of the future will be able to do good work later on in life than those whose energies have been attenuated by anxiety for their old age. The influence of a reasonably assured future upon the mind of the worker is, therefore, an influence making for health and well-being, and that being so, it is our duty to foster the growth of that influence alike as individuals and in our corporate capacity as a society n which every member is interdependent.

